# Peer Review of “Impact of the COVID-19 Pandemic on Routine Childhood Vaccination Coverage in Ecuador From 2019 to 2021: Comparative Analysis”

**DOI:** 10.2196/84847

**Published:** 2025-10-17

**Authors:** Ziqing Wang

**Affiliations:** 1 Columbia University, New York, NY, United States

**Keywords:** COVID-19 pandemic, vaccination coverage, Ecuador, immunization, routine vaccination, health disparities, vaccine hesitancy

*This is the peer-review report for “Impact of the COVID-19 Pandemic on Routine Childhood Vaccination Coverage in Ecuador From 2019 to 2021: Comparative Analysis.”*

## Round 1 Review

### General Comments

This paper [1] presents some descriptive statistics on routine childhood vaccination coverage before and after the COVID-19 pandemic started in Ecuador. The authors found an overall declining trend in routine childhood vaccination coverage since the pandemic started. The aim of the paper is important, but the manuscript seems incomplete. For example, the authors mentioned geographical disparities in access to vaccines but did not clearly present relevant data analysis results to support the statements. Moreover, the authors mentioned that COVID-19 vaccination coverage in the general population was assessed, but they did not include such information in the manuscript.

### Specific Comments

#### Major Comments

1. Please complete the manuscript by adding the results and interpretation of the Joinpoint regression analyses. The authors claimed that Joinpoint regression analyses were conducted but did not present and discuss the results. More importantly, please note that the Joinpoint analysis requires at least 7 time points. The authors only included 3 time points (2019, 2020, 2021). I suggest either calculating vaccination coverage percentages for at least 7 years to run the Joinpoint analysis or just presenting the descriptive statistics for each year without using the Joinpoint analysis.

2. There is a figure that plots the vaccination coverage rates in 2019, 2020, and 2021, but the authors did not provide any description or interpretation of the figure.

3. Please add descriptions for all tables and figures.

4. Please be more specific in the Data Analysis section; for example, please clearly mention what was meant by trend analysis and comparative analysis and include any specific descriptive summaries and/or statistical tests you used.

5. Please improve the organization of the Results section. For example, the regional disparities were discussed at the end of the section, yet they were presented in the first table.

6. Please narrow the focus of the manuscript. It seems that the authors aim to characterize the changes in routine childhood vaccination before and after the COVID pandemic, but in the manuscript, the authors also mention the disparities in getting the COVID-19 vaccine among the entire Ecuador population. These seem like relatively separate topics and could possibly be studied in two manuscripts.

7. Please support all claims with data or citations. For example, if the authors decide to also study the disparities in COVID-19 vaccine access, please include relevant data analysis results in the manuscript.

#### Minor Comments

8. At the start of the Data Analysis section, please cite the specific software used.

9. I was wondering if there is data from after the pandemic (2022 and beyond), so the authors can examine whether routine childhood vaccination coverage went back up or kept declining.

10. Please clarify what Table 1 presents and why it is included.

## References

[1] Sanchez J, Rodriguez AA, Cuello KPM (2025). Impact of the COVID-19 Pandemic on Routine Childhood Vaccination Coverage in Ecuador From 2019 to 2021: Comparative Analysis. JMIRx Med.

